# Expression of Selected Genes Involved in Neurogenesis in the Etiopathogenesis of Depressive Disorders

**DOI:** 10.3390/jpm11030168

**Published:** 2021-03-01

**Authors:** Katarzyna Bliźniewska-Kowalska, Piotr Gałecki, Janusz Szemraj, Monika Talarowska

**Affiliations:** 1 Department of Adult Psychiatry, Medical University of Lodz, 91-229 Lodz, Poland; piotr.galecki@umed.lodz.pl; 2 Department of Medical Biochemistry, Medical University of Lodz, 92-215 Lodz, Poland; janusz.szemraj@umed.lodz.pl; 3 Department of Clinical Psychology, Institute of Psychology University of Lodz, 91-433 Lodz, Poland; talarowskamonika@wp.pl

**Keywords:** neurogenesis, depression, gene expression

## Abstract

(1) Background: The neurogenic theory suggests that impaired neurogenesis within the dentate gyrus of the hippocampus is one of the factors causing depression. Immunology also has an impact on neurotrophic factors. The aim of the study was to assess the importance of selected genes involved in the process of neurogenesis i.e., nerve growth factor (NGF), brain-derived neurotrophic factor (BDNF), glial-derived neurotrophic factor (GDNF) and neuron-restrictive silencer factor (*REST* gene) in the etiopathogenesis of depressive disorders. (2) Methods: A total of 189 subjects took part in the study (95 depressed patients, 94 healthy controls). Sociodemographic data were collected. The severity of depressive symptoms was assessed using the Hamilton Depression Rating Scale (HDRS). RT-PCR was used to assess gene expression at the mRNA levels, while Enzyme-Linked Immunosorbent Assay (ELISA) was used to assess gene expression at the protein level. (3) Results: Expression of *NGF*, *BDNF*, *REST* genes is lower in depressed patients than in the control group, whereas the expression of *GDNF* gene is higher in patients with depressive disorders than in the group of healthy volunteers. (4) Conclusions: The expression of selected genes might serve as a biomarker of depression.

## 1. Introduction

Depression is one of the most common mental disorders. However, its etiopathogenesis is not yet fully understood. One of the biological theories suggests impaired neurogenesis to be a possible cause for the development of this disorder. The neurogenic theory proposes that impaired adult neurogenesis within the dentate gyrus of the hippocampus is one of the factors causing depression and the restoration of normal neurogenesis leads to recovery [1]. This theory is based on three main principles. Firstly, hypercortisolemia impairs the process of neurogenesis and, at the same time, contributes to the development of depression [1,2]. The hippocampus as part of the limbic system is considered to be the so-called “emotional brain” and also the area where the process of adult neurogenesis (AHN) takes place. It is a frequently studied structure in the investigation of depressive disorders. The hippocampus contains many receptors for glucocorticosteroids, is involved in the action of the hypothalamic-pituitary-adrenal axis, which makes it more prone to experiencing stress and depressive symptoms [3]. Glucocorticosteroids affect the process of neurogenesis, both directly and indirectly, by activating pro-inflammatory genes and by reducing the expression of main regulators of neurogenesis, i.e., brain-derived neurotrophic factor (BDNF) [4,5]. Another evidence that supports this theory is a fact that depressed patients have, on average, a smaller volume of the hippocampus [6]. A decrease in synaptic density within the hippocampus correlates with the severity of depressive symptoms [7]. Scientific data confirm that larger hippocampal volumes indicate faster recovery in people suffering from depression [8]. It has also been hypothesized that antidepressants require enhancement of neurogenesis in order to induce their therapeutic effect. Moreover, the delay in their action coincides in time with the time it takes for new neurons to mature and integrate into the hippocampus [1]. Modern fast-acting antidepressants used in treatment-resistant depression (TRD), such as esketamine, also increase signaling by neurotrophic factors, e.g., BDNF, influencing neurogenesis [9].

The process of neurogenesis is regulated by many factors. Neurotrophins, which include, among others, nerve growth factor (NGF), brain-derived neurotrophic factor (BDNF), and glial-derived neurotrophic factor (GDNF), are substances that affect the growth and development of new neurons as well as the survival of existing neuronal cells [10]. The process of neurogenesis is also influenced by transcription factors such as Neuron-Restrictive Silencer Factor (NRSF). NRSF, also known as RE1-Silencing Transcription factor (REST) is one of the main suppressors of the neurogenesis process. It inhibits the expression of neural genes. Deletion of the *REST* gene or functional inhibition of the protein in non-neuronal tissues leads to erroneous expression of neuronal genes and embryonic mortality, while ectopic expression of REST in the nervous system inhibits the expression of neuronal genes and causes the developmental dysfunction. Therefore, REST is important in determining whether a cell has a neuronal phenotype [11,12].

Thus, neurogenesis and factors affecting it remain important in understanding the etiopathogenesis and treatment of depression.

The aim of the study was to evaluate the mRNA and protein expression of selected genes (*NGF*, *BDNF*, *GDNF*, and *REST*) involved in the process of neurogenesis in patients with depressive disorders (study group) and healthy individuals (control group) and to determine the correlations between the gene expression, severity of depression and sociodemographic variables.

## 2. Materials and Methods

### 2.1. Subjects and Data Collection

A total of 189 subjects took part in the study. The study group included 95 patients (67 F, 28 M) with the diagnosis of a depressive episode or recurrent depression disorder (F32 and F33, respectively, according to ICD-10 criteria) [13]. The control group consisted of 94 healthy volunteers (66 F, 28 M) with a negative history for mental disorders. Table 1 shows statistical characteristics of both studied groups. The exclusion criteria were as follows: other psychiatric diagnoses than depressive disorders, serious neurological or somatic diseases that could affect the expression of selected genes, abuse, and addiction to psychoactive substances.

Individuals taking part in the experiment were native Poles from central Poland (not related). They were chosen for the study group at random without replacement sampling. Participation in the study was voluntary. Written informed consent for participation was obtained from each subject according to the study protocol that had been approved by the Bioethical Committee of the Medical University of Lodz (No. RNN/127/17/KE +KE/1446/18). Participation in the study was not associated with any specific changes in the antidepressant therapy.

### 2.2. Hamilton Depression Rating Scale (HDRS)

**Figure 1:**
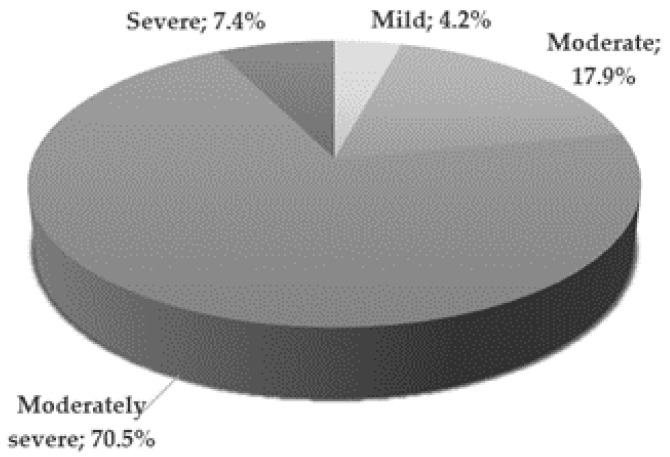
Severity of depression in the study group according to HDRS score criteria; Mild—8–12; Moderate—13–17; Moderately severe—18–29; Severe—≥30 points.

The mental state of patients from the study group was assessed on the day of inclusion in the study by a qualified psychiatrist. The severity of depressive symptoms was assessed using the 17-item Hamilton Depression Rating Scale [14]. Figure 1 illustrates the severity of depression in the study group [15]. Most patients (70.5%) presented moderately severe depressive symptoms.

A statistically significant difference was found between the mean HDRS scores in men and women in the study group (*p* < 0.01). It turned out that the severity of depression in women was greater than in men, because the mean HDRS was significantly higher. The mean values for women and men were respectively: 22.3 ± 4.96 vs. 18.9 ± 4.87 (Table 1).

The peripheral venous blood samples were taken from all the participants. In the study group blood was collected at the beginning of hospitalization, when the depressive symptoms were most severe, before the start or modification of existing antidepressant treatment. RT-PCR was used to assess gene expression at the mRNA level, while ELISA was used to assess the expression at the protein level. The obtained results were subjected to statistical analysis in order to determine the correlation between gene expression and clinical and sociodemographic data.

### 2.3. Collection and Storage of Blood Samples

Blood was collected using an evacuated tube system Becton-Dickenson Vacutainer^®^ with interchangeable glass tubes with ethylenediaminetetraacetic acid (EDTA) as an anticoagulant. Blood samples were fractionated to obtain mononuclear leukocytes and plasma before being analyzed or stored. The extraction of RNA from peripheral blood mononuclear cells (PBMCs) was carried out using commercially available spin column kits (GenElute Mammalian Total RNA Miniprep Kit, Sigma-Aldrich, St. Louis, MO, USA; ISOLATE II RNA/DNA/Protein Kit, Bioline, Alvinston, Canada, respectively) according to the manufacturer’s instruction. Mononuclear cells and plasma were transferred directly to −20 °C. Biological samples were defrosted before the experiment.

### 2.4. Determination of mRNA Gene Expression

#### 2.4.1. Total RNA Isolation

Total RNA isolation from the patients’ blood was performed using InviTrap Spin Universal RNA Kit (Stratec molecular, Berlin, Germany) based on the manufacturer’s recommendations. 300 µL of blood in a test tube was incubated with 300 µL of Lysis/Binding Buffer. 300 µL of Acid Phenol:Chloroform mixture was added to the cellular lysate and, after mixing, the sample was centrifuged (5 min, 10,000× *g*) to separate the aqueous phase from the organic phase. The upper fraction (aqueous) was moved to a fresh test tube, which contained 375 µL of 96% ethanol and, after mixing, the entire content was poured into a test tube with a column and a filter. After centrifugation (15 s, 10,000× *g*), the column with the filter was moved to fresh test tubes and rinsed in 700 µL of RNA Wash Solution 1, and then subjected to centrifugation (10 s, 10,000× *g*). The column with the filter was rinsed twice in 500 µL of Wash Solution 2/3 and centrifuged (1 min, 10,000× *g*). The column with the filter was placed in a fresh test tube and isolated RNA was subject to elution in 30 µL of water free from nucleases (temperature of 95 °C) by means of centrifugation (30 s, 10,000× *g*). Absorbance was measured using a spectrophotometer (Picodrop™ Microliter Spectrophotometer VWR International, LLC, Radnor, PA, USA) at λ = 260 nm in order to determine total RNA concentration. Isolated RNA was stored at −70 °C.

#### 2.4.2. Quality Analysis of Isolated RNA

The quality of total RNA was checked with Agilent RNA 6000 Nano Kit (Agilent Technologies, Santa Clara, CA, USA) in accordance with the manufacturer’s recommendations. 1 µL of RNA 6000 Nano dye was added to a test tube containing 65 µL of Agilent RNA 6000 Nano gel matrix and then centrifuged (10 min, 13,000× *g*). The gel-fluorescent dye mixture was applied on the surface of a Nano chip placed in the workstation. Then, 5 µL of RNA Nano marker was added to selected pits. Isolated samples of RNA and RNA size marker were subject to denaturation (2 min, 70 °C), and then 1 µL of the sample was pipetted to selected pits of the Nano chip and mixed (1 min, 2400 rpm). The quality of isolated RNA was checked using 2100 Bioanalyzer (Agilent Technologies). The level of degradation of total RNA was determined with the use of an electrophoretogram and RNA integrity number (RIN) values recorded. Only the samples with RIN (RNA integrity number) value > 7 were subject to further analysis. All RNA samples had RIN value greater than 7.

#### 2.4.3. RT-PCR Reverse Transcription

RNA was reverse-transcribed into complementary DNA (cDNA) according to the manufacturer’s instructions supplied with the TaqMan^®^ RNA Reverse Transcription Kit (Applied Biosystems, Foster City, CA, USA). The reaction of cDNA synthesis consisted of nuclease-free water, 10× RT Random Primers, 25× dNTP Mix (100 mM), 10× RT Buffer, Reverse Transcriptase, and total RNA (0.5 ng/µL) and was performed using a Biometra TA advances thermocycler (AnalyticJena, Jena, Germany). The reverse transcription included three steps: (i) enzyme activation (30 min at 16 °C), (ii) proper synthesis of cDNA (30 min at 42 °C), and (iii) enzyme inactivation (5 min at 85 °C). The obtained cDNA was stored at −20 °C.

#### 2.4.4. Real-Time PCR Reaction

Real-Time PCR reaction was conducted using TaqMan^®^ Universal PCR Master Mix, No UNG (Applied Biosystems) using TaqMan^®^ RNA Reverse Transcription Kit (Applied Biosystems) based on the manufacturer’s recommendations, using specific Hs00171458_m1, Hs03298540_m1, Hs01931883_s1, Hs05028212_s1, Hs04194366_g1probes, respectively for NGF, *BDNF*, *GDNF*, *REST* and *RPL13A* genes, delivered by Applied Biosystems. according to the protocol provided by the manufacturer. To calculate relative expression of mRNA genes, the Ct comparative method was used [16]. The level of *NGF*, *BDNF*, *GDNF*, *REST* gene expression in particular tissues was normalized in relation to *RPL13A* reference gene.

Each target probe was amplified in a separate 96-well plate. All samples were incubated at 50 °C for 2 min and at 95 °C for 10 min, and then cycled at 95 °C for 30 s, at 60 °C for 30 s and at 72 °C for 1 min; 40 cycles were performed in total. Fluorescence emission data were captured and mRNA levels were quantified using the critical threshold (Ct) value. Analyses were performed with ABI Prism 7000 (Sequence Detection System Software-Applied Biosystems, Foster City, CA, USA). Control samples without RT and with no template cDNA were performed with each assay. Relative gene expression levels were obtained using the ∆∆Ct standard 2−∆∆ct calculations and expressed as a fold change of the control sample [16,17]. Amplification specific transcripts were further confirmed by obtaining melting curve profile.

### 2.5. Determination of NGF, BDNF, GDNF and REST (NRSF) Protein Expression

#### 2.5.1. Determining Protein Concentration

150 µL of the reaction mixture was added to pits containing 150 µL of serum, diluted 10 times in 10 mM of phosphate buffered saline, pH 7.4, and incubated (2 h, 37 °C). To specify protein concentration, an analytical curve for serum albumin was determined. Both the examined samples and the reference samples were made parallel in three repetitions. Sample absorbance was measured using Multiskan Ascent Microplate Photometer (Thermo Labsystems, Philadelphia, PA, USA) at λ = 562 nm and total protein concentration was calculated from the standard curve equation.

#### 2.5.2. Enzyme-Linked Immunosorbent Assay (ELISA)

The concentration of proteins NGF, BDNF, GDNF, REST in the serum of the patients was determined using Human NGF Elisa Kit (MyBiosource, San Diego CA, USA), Human BDNF ELISA Kit (Merck KGaA, Darmstadt, Germany), Human GDNF ELISA Kit (MyBiosource, San Diego CA, USA), Human REST ELISA Kit Biorbyt Exolore Bioreagents (St Louis, MO, USA) according to the protocols provided by the manufacturer. β-actin was used for endogenous control of protein concentration in the samples and determined with the help of Human Actin Beta (ACTb) ELISA Kit (BMASSAY, HaiDian District, BeiJing China) based on the manufacturer’s recommendations. 100 μL of serum (ρprotein = 0.5 mg/mL) was added to pits coated with antibodies specific for the analyzed proteins and incubated (1.5 h, 37 °C). The content was removed and the pits were rinsed three times in 10 mM of phosphate buffered saline and incubated (1 h, 37 °C) with 100 μL of biotinylated antibodies specific for the analyzed proteins. Then, the content was removed and the pits were rinsed three times in 10 mM of phosphate buffered saline and incubated (30 min, 37 °C) with 100 μL of ABC Working Solution. The content was removed and the pits were rinsed five times in 10 mM of phosphate buffered saline and incubated (10 min, 37 °C) with 90 μL of TMB (3,3′,5,5′-Tetramethylbenzidine) substrate. After adding 100 μL of TMB Stop Solution, the absorbance of the samples was measured using Multiskan Ascent Microplate Photometer (Thermo Labsystems, Philadelphia, PA, USA) at λ = 450 nm. To determine protein concentration, analytical curves were made for the analyzed proteins.

### 2.6. Statistical Analysis

A statistical analysis of the material was performed. The qualitative characteristics of the groups of affected patients and healthy controls were expressed as frequencies and shown as percentages. An arithmetical mean ($\bar{X}$) was calculated in order to characterize the average values of quantitative features. Statistical dispersion measures included the values between the minimum and the maximum and standard deviation (SD). The consistency of the distributions of the analyzed quantitative variables with normal distribution was tested using the Shapiro-Wilk test. The distributions differed significantly from the normal distribution, so non-parametric tests were used to compare the means. The Mann-Whitney test for independent samples was used to compare the means of individual variables in the study group and in the control group. Spearman’s rank correlation coefficient was used to assess the relationship between quantitative variables and its significance was assessed by the Student’s t-test. The chi-square test of independence was performed to compare the incidence among men and women in the study and the control groups.

The differences between the means and the relationships between the variables for which the calculated test value was equal to or greater than the critical value read from the appropriate tables with the appropriate number of degrees of freedom and error probability *p* < 0.05 were considered statistically significant.

## 3. Results

### 3.1. Mean Gene Expression

**Figure 2:**
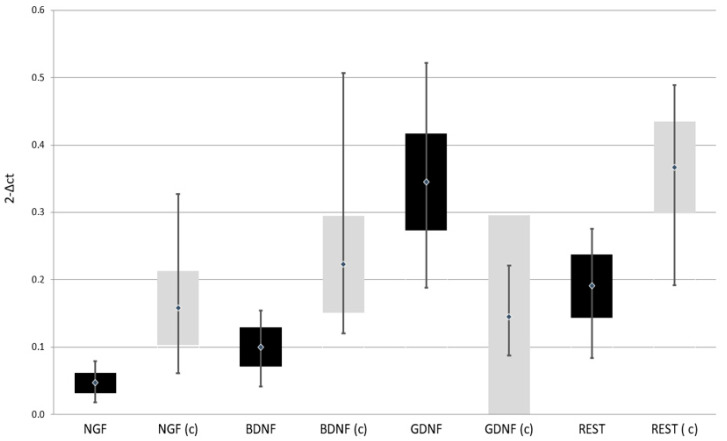
Expression of selected genes regulating the process of neurogenesis at mRNA level in the study and the control group; black—study group; light gray (c)—control group; NGF—Nerve Growth Factor; BDNF—Brain-Derived Neurotrophic Factor; GDNF—Glial-Derived Neurotrophic Factor; REST—RE1-Silencing Transcription factor.

**Figure 3:**
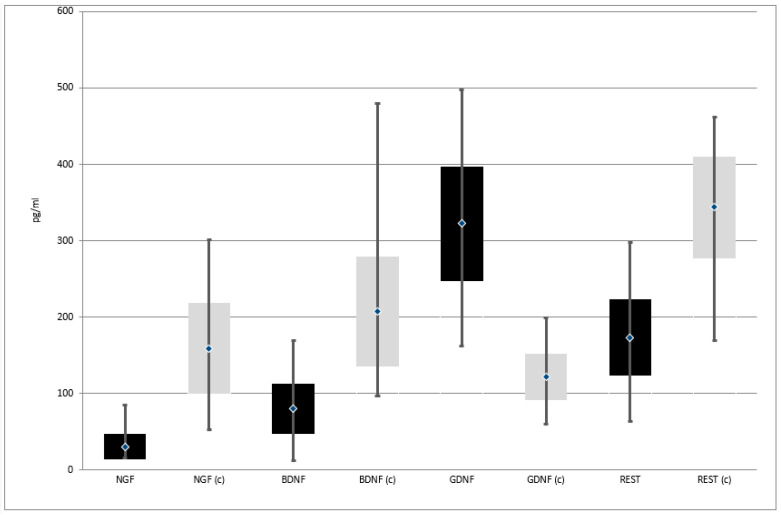
Expression of selected genes regulating the process of neurogenesis at protein level in the study and the control group; black—study group; light gray (c)—control group; NGF—Nerve Growth Factor; BDNF—Brain-Derived Neurotrophic Factor; GDNF—Glial-Derived Neurotrophic Factor; REST—RE1-Silencing Transcription factor.

Expression of *NGF*, *BDNF*, and *REST* genes at both protein and mRNA levels was lower in patients with depressive disorders than in the control group. On the other hand, the expression of *GDNF* gene at both protein and mRNA levels was higher in patients with depressive disorders than in the control group (Table 2, Figure 2 and Figure 3)

### 3.2. Correlation with Depression Severity

In the group of depressed patients, there was analyzed the relationship between mRNA and protein expression for selected genes modulating the neurogenesis process and the severity of depressive symptoms as measured by the HDRS score. There was no statistically significant dependence of HDRS scores on the expression at the mRNA level and also at the protein level for any of the analyzed genes. In each case, the rank correlation coefficient, measuring the strength of the relationship, was very close to zero (Table 3).

### 3.3. Correlation with Gender and Age

Comparison of the expression of all selected genes in the investigated men and women in the study group did not show any statistically significant difference (*p* > 0.05). It turned out that the means for both groups of patients were very similar. The largest differences, although also statistically insignificant, occurred in the case of the REST gene both at the mRNA level: 0.204 ± 0.038 and 0.185 ± 0.050 and at the protein level: 184.5 ± 36.6 and 167.9 ± 54.0. As can be seen, in both cases the average for the surveyed men is higher than for the surveyed women.

The comparison of gene expression in male and female subjects in the control group showed only one statistically significant difference. It turned out that a statistically significant difference occurred in the case of the NGF gene at the protein level (*z* = 2.265; *p* = 0.0235). In this case, the average expression of the NGF gene at the protein level was significantly lower in men than in women: 140.7 ± 68.6 vs. 166.8 ± 53.5. Also in the case of the NGF gene expression at the mRNA level, the difference between the mean values in the group of men and women was significant, but not statistically significant: 0.146 ± 0.067 and 0.163 ± 0.049, respectively. In the case of the expression of the remaining genes, no statistically significant difference was found between the means obtained in men and in women (*p* > 0.05), the means turned out to be quite similar.

Examination of the correlation between the expression at the mRNA level and at the protein level for all selected genes and the age of patients in the study group showed no statistically significant correlation between these variables (*p* > 0.05). The rank correlation coefficients for all assessed relationships were very close to zero, which proves the lack of influence of age on the expression of particular genes.

When the correlation between the expression at the mRNA level and at the protein level for all selected genes and the age of patients in the control group was tested, a statistically significant correlation was demonstrated between the expression of the NGF gene at the protein level and the age of the patients. It was a weak positive correlation, i.e., the older the patient’s age, the greater the expression of this gene (the rank correlation coefficient is 0.235; *p* < 0.05).

The correlation between the age of the patients and the expression of REST at the mRNA level also turned out to be quite close to statistical significance (the rank correlation coefficient is 0.202; *p* = 0.051). This time, the older the respondents age, the greater the expression of this gene.

For all other assessed dependencies, the rank correlation coefficients were close to zero, which proves that age did not influence the expression of particular genes. Although patients from the study group were significantly older than healthy controls, age seems not to play an important role in the expression of selected genes.

## 4. Discussion

### 4.1. Nerve Growth Factor (NGF)

The first discovered neurotrophin is the Nerve Growth Factor (NGF). Our study indicated that in depressed patients, the expression of this factor both at the mRNA level and at the protein level was significantly lower than in the control group of healthy individuals. A meta-analysis performed by Chen et al., which included seven studies comparing peripheral NGF in depressed patients and healthy subjects, similarly to this study, found that NGF levels were significantly lower in depressed patients than in healthy subjects and that this had an inverse correlation with mean age and disease severity. Furthermore, a meta-analysis of four articles found that peripheral NGF levels did not change significantly before and after antidepressant treatment. That meta-analysis also presents quite a controversial conclusion. Namely, in meta-regression, the levels of peripheral NGF showed an inverse correlation with the HAM-D score (HDRS) [18]. In our study, no relationship was found between NGF expression and the severity of depressive symptoms as measured by the HAM-D score (HDRS). Liu et al. suggested that peripheral NGF levels might be more related to stress than to the depressive symptoms themselves [19]. It can be assumed that the NGF level response to stress would be increased acutely through the compensatory effect of HPA axis hyperactivity and cortisol release, but the associated cortical atrophy under pathological chronic stress and chronic HPA axis hyperactivity, for example in depression, would result in a long-term reduction in NGF levels [20,21,22,23]. It is difficult to state unequivocally whether peripheral NGF may be only the result of stress response in depression, or whether it is an etiological factor of these mood disorders. The studies also do not find whether there is a significant difference between the level of nerve growth factor in the serum of patients with depression without and with suicidal thoughts [24]. In the study, the mean expression of the NGF gene at the protein level in the control group was significantly lower in men than in women. Also in the case of NGF gene expression at the mRNA level in the control group, the difference between the mean in the group of men and women was significant, but not statistically significant. No such relationship was observed in the group of depressed patients. In the study by Martocchia et al. examining the concentration of NGF protein in the serum by ELISA method, the values were lower in women than in men. Moreover, the difference in serum NGF concentrations between the follicular and luteal phases in each woman was statistically significant. The differences in NGF concentrations between men and women (in both phases of the menstrual cycle) were also statistically significant. Summarizing, the article suggests the role of sex steroids as modulators of NGF secretion in humans [25]. In the control group of our research there was a statistically significant correlation between the expression of NGF gene at the protein level and the age of the patients. It is a weak positive correlation, i.e., the older the patient’s age, the higher the expression of this gene. In a rat study, elevated levels of proneurotrophin, proNGF and decreased levels of mature NGF were found in the prefrontal cortex and hippocampus in older rats (compared to young controls). The results of this study support the argument that NGF signaling is altered in the aging brain and that such changes may contribute to age-related cognitive decline [26]. Learning and memory disorders in Alzheimer’s disease (AD) are associated with the degeneration of primary forebrain cholinergic neurons (BFCNs). BFCNs extend their axons into the hippocampus, where they bind nerve growth factor (NGF), which is transported back into the cell body. While NGF is essential for BFCN survival and function by binding to the high affinity TrkA receptor, it has been proposed that its unpurified precursor, pro-NGF induces neurodegeneration by binding to p75NTR. NGF levels decline with age, while pro-NGF levels increase. Data from Fortess et al. suggest that the increase in p75NTR from AD may be due to elevated pro-NGF levels as a result of decreased cleavage / conversion and that pro-NGF may be partly responsible for the age-related degenerative changes seen in the primary forebrain [27]. NGF also play a role in immunity. It may influence B-cell as well as T-cell function and particularly plays a role in macrophage migration into inflamed lesions [28]

### 4.2. Brain-Derived Neurotrophic Factor (BDNF)

Brain-Derived Neurotrophic Factor (BDNF) is another important factor modulating the process of neurogenesis. In animal model studies, chronic stress and depressive states decreased BDNF expression, increased apoptosis and decreased neuronal regeneration in the hippocampus, as well as decreased BDNF expression in other parts of the brain [29,30]. Human studies show that brain-derived mature neurotrophic factor (BDNF) (but not its precursor proBDNF) is reduced in patients with depressive disorders [31]. The results of the meta-analysis by Sen et al. provide strong evidence suggesting that serum BDNF levels are low in patients suffering from Major Depressive Disorder (MDD) and that these levels rise after the end of antidepressant treatment. Moreover, big research studies by Bus and Molendijk et al. clearly indicate that decreased serum level of BDNF is characteristic for depression. It is present during the acute phase, but not in full remission [32,33]. BDNF can potentially be used as a biomarker of mental disorders or as a predictor of antidepressant efficacy [34]. Post-mortem studies on depressed patients who committed suicide show that the expression of BDNF and its receptor TrkB decreased [35]. However, the question arises whether there is a relationship between the concentration of BDNF in the serum and its concentration in the CNS. Serum BDNF is derived from megakaryocytes or platelet precursors [36]. However, it can be concluded that it is stored in platelets rather than produced. It can be released into the serum upon activation [37]. BDNF also has the ability to cross the blood-brain barrier [38], which suggests that its serum concentration may correspond to its concentration in the central nervous system (CNS). Karege et al. showed a positive correlation between the concentration of BDNF in the serum and the cerebral cortex in studies on rats. Peng et al. suggested that BDNF can be used as an additional test to monitor the effectiveness of repetitive Transcranial Magnetic Stimulation (rTMS) treatment in patients with depression [39]. Reduced BDNF levels may have an impact on the development of depression in patients with Parkinson’s disease [40]. Bus et al. suggested that depressive episodes contribute to the decrease in serum BDNF rather than lowered BDNF to the onset of depression [32]. In our research, no statistically significant correlation was observed between BDNF expression and the severity of depression symptoms measured with the HDRS scale, which confirms the conclusions of the study by Jevtović et al. [41] and Caldieraro et al. [42] who also found no relationship between the severity of depression and serum BDNF concentration. However, several studies showed a relationship between the severity of depression symptoms and the concentration of BDNF [43,44,45] presenting it as a negative correlation [46]. Lang et al. studied 118 healthy volunteers. Based on this study, they concluded that low BDNF levels in healthy subjects with depressive personality traits may be a risk marker, reflecting the personality profile that is associated with susceptibility to mood disorders [47]. In our study, no correlation was found between BDNF expression and age, gender of the participants. Lommatzsch et al. reported that plasma BDNF levels decreased significantly with age, and women showed significantly lower platelet BDNF levels than men. Moreover, plaque BDNF levels changed during the menstrual cycle [48]. Chan and Ye in their review emphasized the importance of gender, suggesting BDNF content in certain parts of the brain and the tendency to develop BDNF-deficient diseases, such as depression, to be higher in females. Sex hormones or steroids can modulate BDNF activity. For example, estrogen has a positive regulatory effect on BDNF expression and signaling [49]. Inflammatory factors, cytokines, reduce the expression of BDNF, subsequently leading to a decrease in neurogenesis. On the other hand, the immunomodulatory process also requires the regulation of signaling pathways mediated by BDNF. This role of BDNF in the regulation of the neuroimmune axis is emphasized in the work of Jin et al. [50]. Neurotrophic factors may modulate immune responses [28].

### 4.3. Glial-Derived Neurotrophic Factor (GDNF)

In a meta-analysis conducted by Lin and Tseng [51] assessing the concentration of glial neurotrophic factor (GDNF) in patients with depression, it was confirmed that in this group of patients the blood levels of GDNF are significantly reduced compared to healthy subjects. In our research, we observed the opposite relationship. GDNF expression at both the protein and mRNA levels was higher in depressed patients than in the control group. A study by Park and Lee [52] showed higher levels of GDNF in patients with mild to moderate depressive episode before antidepressant treatment than afterwards. The systematic review of 2016 [53] also noted this inconsistency in the results. Most studies found a reduction in GDNF levels in depressed patients compared to the healthy group. However, Wang et al. [54] found an increase in the GDNF level in the plasma of depressed patients compared to controls. Michel et al. [55] described an increase in the level of GDNF in homogenates of the parietal cortex of deceased patients with depression. Treatment can affect GDNF levels in various brain structures. Lithium administered to rats increased the concentration of GDNF in the prefrontal cortex and lowered its concentration in the hippocampus [56]. Patients in the study group received antidepressant treatment, which may explain the increased level of GDNF, but further research is necessary to explain this phenomenon.

### 4.4. RE1-Silencing Transcription Factor (REST)/Neuron-Restrictive Silencer Factor (NRSF)

There is growing evidence that abnormal transcriptional regulation is one of the key components of the pathophysiology of mood disorders. The Neuron-Restrictive Silencer Factor is a negative regulator of genes that contain the binding site of the repressor element 1 (RE-1) [57]. NRSF is encoded by the *REST* gene. NRSF has multiple target genes including corticotrophin releasing hormone (CRH), brain-derived neurotrophic factor (BDNF), and serotonin receptor 1A, suggesting that it is involved in the pathophysiology of depression. However, the potential role of NRSF-mediated transcriptional regulation in mood disorders remains unclear. In their study, Otsuki et al. [58] assessed the levels of REST mRNA and its known and putative target genes using quantitative real-time PCR in the peripheral blood cells of patients with major depressive (MDD) and bipolar disorders (BD), both in acute depressive episode and in remission. They found decreased REST mRNA expression and increased mRNA expression of CRH, adenylate cyclase 5 and the tumor necrosis factor superfamily in patients with major depressive disorder during an acute episode but not in remission. Altered expression of these mRNAs was not found in patients with bipolar disorder. These results suggest that REST-mediated abnormal transcriptional regulation may be associated with the pathophysiology of depression [58]. In this research, a reduced expression of the *REST* gene, both at the protein and mRNA levels was found in depressed patients compared to healthy controls. REST deregulation has been associated with many neurodegenerative disorders and diseases. REST is activated in selectively sensitive mature neurons of the hippocampus in response to ischemic injuries [59,60,61,62,63] and seizures [64,65,66,67]. In aging neurons, REST loss is associated with the onset of Alzheimer’s disease in humans [68]. Interestingly, in our research, there was found a relationship between the age of patients and REST expression at the mRNA level that is quite close to statistical significance, suggesting that the older the age of the respondents, the greater the expression of this gene was. However, this only applied to subjects in the control group, i.e., healthy individuals because in depressed patients, age had no effect on REST expression. REST is responsible for the suppression of genes responsible for the death of neurons, i.e., neuroprotection. In our study, the control group subjects were slightly younger than patients in the study group, which may translate into such a relationship between REST expression and age in this group.

## 5. Limitations

Qualification for participation in the study was based on the ICD-10 diagnostic criteria. A detailed diagnostic interview, performed by experienced psychiatrists, made it possible to minimize the risk of qualifying patients from other groups of mental disorders, including people with a risk of bipolar affective disorders. However, it cannot be ruled out that some study participants in the future will not experience maniac or hippomaniac episodes and therefore, the primary diagnosis will change [69].

Research indicates that peripheral blood determinations are expressed at the mRNA level and at the protein level as genes to a large extent reflect expression in the central nervous system [70]. However, there is no possibility to compare the results for selected genes in the group of patients with depressive disorders in the available literature.

Although patients from the study group were older than healthy controls, age seems not to play an important role in the expression of selected genes. Examination of the correlation between the expression for all selected genes and the age of patients with depression showed no statistically significant correlation between these variables (*p* > 0.05).

## 6. Conclusions

Expression of the NGF, BDNF, and REST genes at both protein and mRNA levels is lower in patients with depressive disorders than in the control group. On the other hand, the expression of GDNF gene at both protein and mRNA levels is higher in patients with depressive disorders than in the control group. Expression of the analyzed genes modulating the process of neurogenesis does not depend on the severity of depressive symptoms, gender, or age of the depressed subjects. Although further research is needed, it can be concluded that the factors involved in the process of neurogenesis may serve as biomarkers of depressive disorders. It is worth mentioning that neurotrophins through their widespread expression also in immune cells are candidate molecules for regulating neuroimmune interactions [71].

## Figures and Tables

**Table 1:** The characteristics of the studied groups.

Variable		Group										Means Comparison
		Study					Control					
Gender		*n*		%			*n*		%			
	M	28		29.5			28		29.8			*chi2* = 0.002; *p* = 0.962;
	F	67		70.5			66		70.2			
		min	max	$\bar{X}$	SD	V(%)	min	max	$\bar{X}$	SD	V(%)	
Age (years)		18	65	43.1	14.1	32.6	22	63	34.6	10.4	30.0	*Z* = 3.961; *p* = 0.0001 *
HDRS score (points)	M	8	28	18.9	4.87	25.7						*Z* = 2.751; *p* = 0.0059 * (between genders)
	F	11	32	22.3	4.96	22.2						

M—male; F—female; HDRS—Hamilton Depression Rating Scale; *n*—number; %—percentage; min-minimum value; max—maximum value; $\bar{X}$—arithmetic average; SD—standard deviation; V (%)—coefficient of variation; *ch2*—*chi2* test result; *Z*—Mann-Whitney test; *p*—statistical significance; *—statistically significant.

**Table 2:** Comparison of neurogenesis regulating genes expression in both studied groups.

Factor	Expression	Group								Mean Comparison
		Study				Control				
		min	max	$\bar{X}$	SD	min	max	$\bar{X}$	SD	
NGF	mRNA (2−Δct)	0.018	0.079	0.047	0.015	0.061	0.327	0.158	0.055	*Z* = 3.961; *p* = 0.0000 *
	protein (pg/mL)	16	85	30.3	16.7	52	301	159.0	59.2	*Z* = 11.787; *p* = 0.0000 *
BDNF	mRNA (2−Δct)	0.042	0.154	0.100	0.029	0.121	0.507	0.223	0.072	*Z* = 11.453; *p* = 0.0000 *
	protein (pg/mL)	12	169	79.6	32.4	97	479	206.9	71.6	*Z* = 11.787; *p* = 0.0000 *
GDNF	mRNA (2−Δct)	0.188	0.522	0.345	0.072	0.088	0.221	0.145	0.030	*Z* = 11.817; *p* = 0.0000 *
	protein (pg/mL)	162	497	322.2	75.0	60	199	121.3	30.3	*Z* = 11.791; *p* = 0.0000 *
REST	mRNA (2−Δct)	0.084	0.276	0.191	0.047	0.192	0.489	0.367	0.068	*Z* = 11.656; *p* = 0.0000 *
	protein (pg/mL)	63	297	172.8	49.9	169	461	343.7	66.6	*Z* = 11.530; *p* = 0.0000 *

NGF—Nerve Growth Factor; BDNF—Brain-Derived Neurotrophic Factor; GDNF—Glial-Derived Neurotrophic Factor; REST—RE1-Silencing Transcription factor (also known as NRSF—Neuron-Restrictive Silencer Factor); min—minimum value; max—maximum value; $\bar{X}$—arithmetic average; SD—standard deviation; *Z*—Mann-Whitney test; *p*—statistical significance; *—statistically significant.

**Table 3:** Correlation between mRNA and protein expression for selected genes regulating the process of neurogenesis and the severity of depressive symptoms measured with HDRS score in patients from the study group.

Correlation Between HDRS Score and	Spearman’s Rank Correlation Coefficient	Student’s *t*-Test Value	*p*
NGF mRNA expression (2−Δct)	−0.051	0.490	0.625
NGF protein expression (pg/mL)	−0.058	0.561	0.576
BDNF mRNA expression (2−Δct)	0.002	0.023	0.981
BDNF protein expression (pg/mL)	−0.071	0.688	0.493
GDNF mRNA expression (2−Δct)	−0.037	0.357	0.722
GDNF protein expression (pg/mL)	0.006	0.058	0.954
REST mRNA expression (2−Δct)	0.072	0.693	0.490
REST protein expression (pg/mL)	0.031	0.295	0.768

*p*—statistical significance; NGF—Nerve Growth Factor; BDNF—Brain-Derived Neurotrophic Factor; GDNF—Glial-Derived Neurotrophic Factor; REST—RE1-Silencing Transcription factor.

## Data Availability

The data analyzed in the study are available upon request to the authors of the article.
